# Emerging Trends and Issues in Geo-Spatial Environmental Health: A Critical Perspective

**DOI:** 10.3390/ijerph22020286

**Published:** 2025-02-14

**Authors:** Daniel A. Griffith

**Affiliations:** Department of Geospatial Information Sciences, the University of Texas at Dallas, 800 W. Campbell Rd., Richardson, TX 75080-3021, USA; dgriffith5@collin.edu

**Keywords:** hierarchy autocorrelation, mixture, omitted variable bias, spatial autocorrelation, urban hierarchy

## Abstract

This opinion piece postulates that quantitative environmental research and public health spatial analysts unknowingly tolerate certain spatial statistical model specification errors, whose remedies constitute some of the urgent emerging trends and issues in this subfield (e.g., forecasting disease spreading). Within this context, this paper addresses misspecifications affiliated with omitted variable bias complications arising from ignoring, and hence abandoning, negative spatial autocorrelation latent in georeferenced disease data, and/or being ill-informed about reigning teledependencies (i.e., long-distance spatial correlations). As imperative academic challenges, it advances elegant and convincing arguments to do otherwise. Its two particular themes are positive–negative spatial autocorrelation mixtures, and hierarchical autocorrelation generated by hegemonic urban systems. Comprehensive interpretations and implementations of these two conjectures constitute future research directions. Important conceptualizations for treatments reported in this paper include confounding variables and Moran eigenvector spatial filtering. This paper’s fundamental implication is an advocacy for a prodigious paradigm shift, a marked change in the collective mindsets and applications of spatial epidemiologists when specifying spatial regression equations to describe either environmental health data, or a publicly transparent geographic diffusion of diseases.

## 1. Introduction

This perception article offers a personal analysis of and expert judgments about two important contemporary geospatial environmental health characteristics while focusing on selected literature published in the past three years. One theme is the presence of positive and negative spatial autocorrelation (SA) mixtures in georeferenced environmentally sourced disease data (i.e., an illness caused or exacerbated by environmental factors, such as polluted air or water, risk-amplifying exposure to harmful chemicals, and/or vector-borne contaminated organisms; [1,2]). A second topic is the existence of non-coincidental, salient, non-contiguous spatial dependencies in geo-tagged data, ones reminiscent of seasonal effects in time series data, reflecting an SA mechanism that fosters spatial correlation jumps through geographic space; i.e., a virtual or teleporting (i.e., teledependency) data manifestation]. Hierarchy—more specifically, urban hierarchy in practice (e.g., [3]), which can be, among other alternatives, retail-, transportation-, and/or governance-oriented—is the operational construct in this latter situation. Therefore, the viewpoint advocated here is that SA is everywhere, saturating georegistrated disease cases and other types of spatial data in a mixture form, jointly channeled by both relatively simple (e.g., juxtaposition) and complex (e.g., tele-translocation) spatial organizations.

## 2. What Is SA?

The initial task for articulating a rational standpoint for this paper is to define SA (see [4]). Not only is this notion, at least tacitly (e.g., a nonverbal awareness of it), approximately a century old, but, during the last half-century, numerous authors have proffered formal definitions for it. A synthesis of these sundry academic labors, whose domain inadvertently has been restricted to contiguous areal unit phenomenon covariations, building upon the classical bivariate attribute correlation idea in traditional statistics (also known as the general Pearson product moment correlation coefficient), yields the following definitional statement:

The average nature and degree of geographically neighboring geo-tagged phenomena—e.g., geographic information system (GIS) attribute values—(dis)similarities for a moving window (i.e., a portable square frame employed to identify a subregion of interest consisting of many areal units when superimposed somewhere on a geographic landscape) sliding across a two-dimensional surface (e.g., a map) such that: (1) phenomena haphazardly arranged across a geographic landscape, void of any map pattern, constitute zero SA (i.e., a random assortment of neighboring phenomena); (2) global map-wide gradient, regional geographic cluster, and/or local geographic coterie arrangements of similar neighboring phenomena interspersed across a geographic landscape portraying a synchronous two-dimensional map pattern constitutes positive SA (i.e., alike phenomena geographically bunching); (3) global, regional, and/or local constellations of juxtaposed contrasting phenomena across a geographic landscape depicting a two-dimensional spatial competition map pattern constitute negative SA (i.e., unalike phenomena geographically clumping); and (4) positive–negative SA mixtures typify many, if not most, geographic distributions of phenomena.

**Figure 1:**
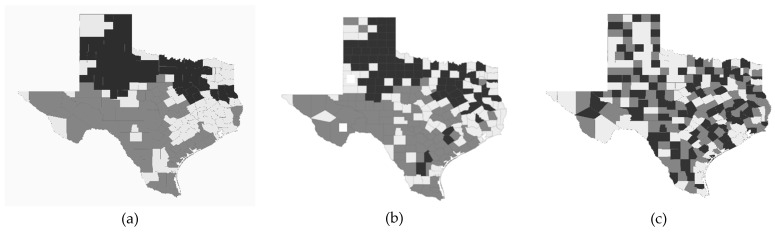
West Nile virus in Texas, 2012-2017; black, gray, and white, respectively, denote relatively high, intermediate, and low numerical values. Left (**a**): positive SA eigenvector spatial filter (ESF). Middle (**b**): composite positive–negative SA mixture ESF. Right (**c**): negative SA ESF.

This description implies that SA originates from one of two distinct redundant information inducers, namely, a common factor—a relentless georeferenced environmental data catalyst—or direct dyadic spatial interaction between phenomena occupying nearby locations—a recurrent spillover effect adulterating independent observations—events in each site, in turn, generate effects in its proximate sites. Griffith [2] furnishes multiple disease mapping examples (e.g. chikungunya, cholera, malaria, tuberculous, and West Nile virus) illustrating prevailing positive SA, negative SA, and positive–negative SA mixtures. Figure 1 is fashioned after one of his specimens. Positive SA describes harmonized attribute values (Figure 1a), engendering a relatively smooth choropleth map portraying relatively few similar value geographic clusters. In contrast, negative SA describes adjacent contrasting attribute values (Figure 1c), begetting a mosaic choropleth map portraying a multitude of high-low/low-high juxtapositions coalescing into geographic clusters. Fusing these two map types, in which positive SA dominates, renders a map like Figure 1b, which displays conspicuous positive SA, usually tempered to be weak-to-moderate (e.g., some of the negative SA map pattern peeks through the positive SA map pattern).

However, SA is not strictly an equal opportunities matter; rather, it tends to be at least partially structured hegemony. Dating back to the Roman Empire, the framework supporting its space-economy structuration frequently is an urban hierarchy. Although the Phoenicians failed to embrace this possibility, imperial Roman expansion engineered it by design. Similar evolutions transpired in other parts of the ancient world, such as China, India, and the Americas (e.g., the Maya civilization), as societies in antiquity progressed from individual city-states concentrated at ports or along rivers, to subjugated inland as well as such inlet settlements, with centralized government at a single location requiring satellite administrations to manage its dauntingly large territory in a pyramidal executive manner. Therefore, the fifth point this preceding SA definition needs to incorporate in order to be properly encyclopedic may be phrased as follows:

(5) as a tree-like graph joining tiers in a branching manner, places populating uninterruptedly linked serviceable geographic (e.g., urban) hierarchy tiers are directly correlated, whereas those having intervening places in their dyad branches are indirectly correlated.

This definitional addition captures spatial dependency leaps across space, tends to be more a function of spatial interaction (e.g., flows of commodities, information, money, people/animals, services, and the such) than common factors (e.g., confounder variables), supplements contiguity-based SA, and immediately relates to geographic diffusion. Unfortunately, because hierarchies tend to comprise tiers stacked to resemble a pyramidal structural shape, a consensus about their effective and efficient scientific visualizations has yet to crystalize. Nevertheless, as Griffith and Li [5] demonstrate, the eigenvectors calibrating hierarchical SA components are appealing surrogates that facilitate substantive missing covariate searches. This spatial statistical tool gap remains an important one in need of future research.

The foregoing social gravity model equational depiction may be stated algebraically as follows:

(1) $$F_{\mathrm{ij}}\;\approx \;\kappa O_{i}^{\alpha }D_{j}^{\delta }e^{-\gamma d_{ij}}\;e^{\rho_{ij}}$$

where F_ij_ denotes the non-negative geographic flow between origin i and destination j locations, O_i_ and D_j_ respectively denote the non-negative magnitudes of a relevant attribute existing at the labeled origin-destination pair (e.g., masses in Newtonian physics), d_ij_ denotes the non-negative metric quantifying the separation between locations i and j (e.g., physical straight-line or great-circle or shortest path distance, time cost, monetary expense, cultural/cognitive dissonance), κ denotes a non-negative constant of proportionality (i.e., a rescaling factor because the arithmetic operation merging O_i_ and D_j_ is multiplication), α and β, respectively, denote non-negative weights whose comparative values are indicative of the relative importance of their assigned categories, γ denotes a distance decay parameter, and ρ_ij_ denotes an origin-destination duad-clique SA effect. Setting aside ρ_ij_ (e.g., imposing ceteris paribus conditions), if α = β = 0, then the materialized SA conforms to the conventional contiguity/neighboring formulation, being strictly a function of d_ij_ and γ. Again, setting aside ρ_ij_ (e.g., imposing ceteris paribus stipulations), if γ = 0, then the materialized SA is solely a function of the importance of origins and/or destinations, and hence corresponds to the geographic hierarchy creation. Finally, the exponent ρ_ij_, absent from original gravity model specifications, represents SA effects such that spatial spillovers inflate flows when ρ_ij_ > 0, deflate flows when ρ_ij_ < 0, and are neutral when ρ_ij_ ≈ 0. Equation (1) appears in the geographic diffusion literature, a body of knowledge in which it enjoys a long-standing history.

This preceding discussion alludes to specific actors or conditions in a particular environment or medical geography dataset that affect the nature and degree to which nearby locations/areal units are (dis)similar to each other in terms of the disease being studied. Environmental pollutants/toxins or deleterious mineral deposits (e.g., uranium, thorium), for example, serve as common factors underlying geographic swaths of illness (e.g., pediatric elevated blood lead levels, radon-induced lung cancer). Government policies and cultural enclaves, for example, tend to create pockets of unvaccinated children, in turn creating hot spots of preventable diseases (e.g., measles). Historical contexts, such as untreated rural well water as a Helicobacter pylori (*H. pylori*) bacterium—routinely transmitted via person-to-person contact but might also be spread through environmental sources—reservoir discovered in 1998 by Pennsylvania State University/Harrisburg researchers (who linked it to stomach ulcers in 1999) in over 75% of surface water samples in Central Pennsylvania. Economic stimuli embedded in historical circumstances generate spatial interactions, such as migration, that urban networks tend to channel. These and other contextual elements can either enhance or diminish SA by affecting how strongly or weakly areal units (like synthetic grid cells, neighborhoods, cities, regions, or countries) correlate with each other.

Given the reality of SA, comprehensively understanding it compels spatial analysts to concede that negative SA is non-fiction instead of fantasy. For a suite of different reasons, some seemingly legitimate, negative SA has been dismissed in practice, with many coherent arguments maintaining that positive SA is the only feasible option. Serendipitously, while demonstrating that negative SA debatably is the most overlooked data feature in spatial statistics, Griffith (e.g., [4]) documents the presence of positive and negative SA mixtures in disease and other georeferenced data. One empirical finding is that this blending chronically has a dominant positive SA component overshadowing its negative accompaniment, resulting in an all too frequent global net weak-to-moderate positive SA index [e.g., Moran coefficient (MC) or Geary ratio (GR)] measure. The collective impression emerging over time is that negative SA is rare, or even illusive. Interestingly, a 2008 study about the mosquito vector spreading malaria in Africa was the first to reveal a positive–negative SA concoction in a formal and authenticated fashion, one in which net SA is almost exactly zero. The alarm sounding here is an absence of any detectable SA in the real world replete with SA (see [1]). Intuitively speaking, SA principally regulates any communicable disease through spatial interaction, and any vector-borne disease via common factors. In doing so, it tends to encapsulate preferentially both centripetal and centrifugal covariations arising from geographic processes.

## 2.1. Quantitatively Indexing SA

The customary indices for gauging SA are the MC and the GR, both of which continue to be ongoing active research subjects (e.g., [6,7,8]). Of the two, the MC is the most prominent SA yardstick, in part because of its overall superior statistical power. With regard to environmental epidemiology, among other medical science specialties, a context within which geospatial data originate from a variety of domains, a persistent data analytic concern has been its vulnerability to the presence of outliers and non-normality, both potentially problematic disadvantages when undertaking a meaningful preliminary/exploratory diagnostic assessment of spatial data, prompting it to remain susceptible to inherent flaws such weaknesses may foster. To this end, Nardelli and Arbia [9], for example, seek to bolster the resilience of these traditional MC and GR calculations.

Two crucial MC ingredients highlighted here for clarity purposes are the spatial weights matrix (SWM) it, and any other spatial statistic, employs, and the algebraic covariation computation it utilizes. A n-by-n binary 0-1 connectivity matrix **C** often denotes this first mathematical construct—a numerical table, analogous to a MSExcel spreadsheet, with n rows and n columns whose intersection cells (denoted by c_ij_) designate which of n^2^ location couplings have direct pairwise correlation. If the areal unit row i and column j twosome represents neighboring areal units, then c_ij_ =1; otherwise, c_ij_ = 0. Because SA is the target estimate, c_ii_ = 0 by fiat. In other words, matrix **C** condenses the geographic configuration/arrangement of a set of n areal units. To dissipate imaginable notational confusion, it is notable that matrix **W** replaces matrix **C** when the latter is row-standardized (i.e., forcing all of the non-negative values in each row to sum to one).

Time series analysis provides many of the earliest scholarly excursions into the autocorrelation realm. Its matrix **C** contains all zeroes except for the upper and lower off-diagonal cells, which contain ones, averaging nearly two ones per row. Matix **C** for spatial series is denser, typically containing four-to-six ones per row, on average. Symbolizing n values of some attribute variable Y in matrix format, say **Y**, and converting this vector to z-scores, yielding **z**_Y_, allows covariation to be parsimoniously written as the following arithmetic means:

$$\mathrm{classical}\;\mathrm{bivariate}:\;z_{Y}^{T}z_{Y}/(n\;–\;1)=z_{Y}^{T}{Iz}_{Y}/(n\;–\;1),$$

where **I** is the n-by-n identity matrix, and superscript T is the matrix transpose operator,

$$\mathrm{SA}:\;z_{Y}^{T}{Cz}_{Y}/n*,$$

where n* predominantly denotes the total of non-zero entries in matrix **C**

This z-score version discloses a connection between these summary statistics and, respectively, a traditional and a Moran scatterplot, two visualization tools useful for portraying their associated Pearson product moment correlation or SA. It also spotlights that, although the first expression has a range of ±1 (e.g., it contains the matrix equivalent **I** = **C**^0^ rather than explicitly **C** itself), the second does not. Another revelation here is that the second expression is unchanged for time and spatial series calculations. On the one hand, this apparent isomorphism suggests the blunder of deducing that what is needed to be known about spatial statistics can be learned from the times series analysis theory. On the other hand, the building blocks of square matrices, namely eigenvalues (essentially scaling factors) and eigenvectors (essentially non-zero vector dimension factors), tell an alternative story, especially because the simplicity of matrix **C** for the time series is almost always lost with spatial series. As and aside, to illustrate these mathematical concepts, choosing a sizeable systematic random sample of three-dimensional coordinates from the space occupied by a dirigible, which is an object elliptical in shape (i.e., and ellipsoid), and then submitting these data to an appropriate eigenfunction analysis yields three eigenvalues and their corresponding eigenvectors. The eigenvectors identify the length, width, and height dimensions of the zeppelin under study, and their accompanying eigenvalues measure the distance from the center of the airship [i.e., (0, 0, 0)] to its hull along each of the three perpendicular axes.

Clearly, the SWM **C** is adaptable for geographic hierarchy articulations, as Griffith and Li [5] corroborate. The compulsory revision for this adjustment parallels that for translating matrix **C** from a time to a spatial series scenario: positing a suitable definition of cell entries c_ij_. In this hierarchical circumstance, if row i and column j have a direct linkage uninterrupted by an intervening element (e.g., city), then c_ij_ =1; otherwise, c_ij_ = 0.

## 2.2. Quantitatively Describing SA

A general tool supplying informative SA descriptions is spatial regression, a refinement of Galton’s originally inaptly named statistical gadget, which takes on various forms. Most notably, there is spatial autoregression (e.g., Besag’s auto models). Because SA mixtures are the topic here, the necessary specification is a two-SA parameter autoregressive-moving average equation—known acronymically as a SARMA model (e.g., see [10,11]), with SAR and MA respectively abbreviating simultaneous autoregressive and moving average—that employs the same SWM for estimating each parameter. The expectation is that one parameter will uncover positive, and the other negative, SA. An ancillary hypothesis is that these two estimates will not covary to such an extent that their outcome implies nothing more than a nonlinear trade-off between them (i.e., the only reason one increases is because the other decreases). The goal is to substantiate the presence of positive–negative SA mixtures, and demystify the empirical rule postulating that, although remotely sensed satellite images embedding fine geographic resolution render 0.9+ SA estimates, disease data tend to deliver SA estimates in the 0.4-0.6 range. But SARMA statistical model theory has the serious shortcoming of being restricted to normal curve theory, either in its pure form or within its Box–Cox power transformation enhancement. Accordingly, generalized linear model (GLM) theory applied to geospatial random variables pleads for a novel spatial statistical regression approach.

In keeping with the prevailing MC guidance tradition, a novel spatial regression [12]–Moran eigenvector spatial filtering (MESF)–is craftable by exploiting the aforementioned eigenfunctions of a modified version of SWM **C**, namely the doubly centered expression present in the MC numerator, written as

(**I** – **11**^T^/n)**C**(**I** – **11**^T^/n), (2)

where **1** denotes an n-by-1 vector of ones. This specification engenders eigenvectors that depict the full spectrum of possible orthogonal and uncorrelated map patterns, with their attendant eigenvalues rescaling to a MC, signifying the nature and degree of the SA an eigenvector portrays; some are positive, whereas others are negative, SA components. The spatial regression includes a linear combination of judiciously chosen (e.g., stepwise selection) eigenvectors (called an ESF), which, in its simplest version, filters SA out of regression residuals and transfers it to the intercept term, switching that term from a constant to a variable [13]. The expectation is that a constructed ESF will decompose into two linear combinations of eigenvectors, one accounting for positive, and the other for negative, SA. The only operational requirement is that a probability model regression computer routine (e.g., linear, lognormal, logistic, multinomial, Poisson, negative binomial, exponential, gamma, beta, inverse Gaussian, Tweedie, Weibull) is available. Hence, this is a necessary tool for decomposing global SA into a positive–negative SA mixture. An ancillary hypothesis is that the sum of these two constituents will be global net moderate positive SA. An affiliated anticipation is that SARMA and MESF findings will be consistent (except plausibly for model misspecification error).

Extending this approach to hierarchy-designed SWMs, in keeping with Griffith and Li [5], eigenvectors extracted from an adjusted edition of that matrix should be germane surrogates for MESF spatial regression.

## 3. SA Mixtures in Disease Data

Anchoring in its cognate health geography/GIScience subdiscipline, disease mapping (e.g., see [14])–two-dimensional (2-D) graphics rendering geographic distribution of diseases map patterns visibly inculcated with SA—is profuse in epidemiology. Not surprisingly, countless public and private health officials, medical researchers, and healthcare professionals and educators are familiar, some more extensively than others, with the presence and workings of SA in the reservoirs, outbreaks, spread/diminution, national/regional/local epi curves (aka epidemic curves), and containment of and vaccinations against both infectious and noncontagious (e.g., vector-borne) sickness waves usually cyclically sweeping across geographic landscapes. This incessant mindfulness and understanding most likely originated in the era of John Snow (arguably the Father of public health) and his infamous Broad Street pump of 1854 London, England. Regardless, its focus has been almost exclusively on positive SA, apart from local SA investigations, which habitually report local MC and Getis–Ord statistics pinpointing isolated positive (e.g., disease hot and cold spots) as well as negative SA geographic clusters of adjacent contrasting attribute values (e.g., spatial outliers). This piecemeal meta-collection of evidence attests to the proposition that positive–negative SA mixtures permeate many georeferenced datasets, albeit a comprehensive inventory of thematic data types has yet to be explored.

Although this mixture conceptualization is nearly two decades old, being predated by the perplexing discovery of hidden negative SA (i.e., net positive SA plays a masking role, typically disguising a lesser negative SA magnitude) in 2006, the acceptance and consequences of the idea that negative SA tends to be an unlikely companion of positive SA are yet to be even modestly embraced. Widespread resistance exists to accept the belief that real-world negative SA is not an artifact of methodology, for example, either by itself or in blendings. A contemporary campaign to debunk this myth already shows empirical examples of positive–negative SA mixtures in a large percentage of geospatial socio-economic/demographic, agricultural, and disease data ([2]; documenting more substantive datasets remains an unfinished state-of-the-art task). Table 1, whose recordings epitomize a purposeful sample covering a wide assortment of Earth locations and geographic resolutions, accentuates this preceding epidemiological case, somewhat integrating as well as supplementing, but not truly duplicating, a reported tabulation in [2]. Its contents entail several illuminating data qualities. Foremost is that a preponderance of, but not all, geo-tagged datasets contain(s) positive–negative SA mixtures. The literature recounts incidents in which SA latent in socio-economic/demographic data endorses this claim. Second, as already conjectured, positive SA unremittingly tends to dominate negative SA (e.g., the weaker MA, instead of the stronger SAR, SARMA component tends to account for it). The MESF computations bolster this interpretation by insinuating that variance inflation may be attributable to positive SA alone. Third, the SARMA autoregressive term is capable of achieving positive SA levels in the 0.9+ range, similar to those lurking about in remotely sensed data, dispelling another misconception about the near-ubiquity of moderate positive SA degrees in such non-satellite sourced attribute datasets.

In conclusion, this perspective article promotes the analytical postulate that not only does moderate-to-strong or very strong positive SA tend to ingrain itself in geospatial disease data (which, when combined with prevailing negative SA most likely renders a net global weak-to-moderate positive level), but negative SA also is a regular hallmark of such data. Furthermore, positive SA may well be inconspicuously stronger than a single global MC and/or GR index value signals. An instinctive corollary is the need for more empirical and simulation research to broaden the scope of the Table 1 entries. In addition, comparative differences between negative SA estimates in the linear–nonlinear class of models imply that misspecification can severely affect latent degree inferences.

## 4. Hierarchical SA in Disease Data

Motivated by the pressing world calamity, as academicians rushed to address, in real time, describing and forecasting the COVID-19 spread in its 2020 pandemic, their plethora of publications fell short by almost unanimously neglecting the international, national, regional, and local urban hierarchy proliferation apparatuses guiding it. Table 2 illustratively approximates this dire oversight that provoked Griffith and Li [5] to a proactive leadership engagement rectifying what otherwise was a lost opportunity, acknowledging the detrimental impact of blindness to the influence of geographic hierarchies on efficaciously handling and mastering the pandemic in question, and implementing steps to correct this misplaced puzzle piece with a penned proposal of their solutions and groundwork output. Although this evidenced-based opinion may be debatable, and hence constitutes a topic for future research, it is consistent with classical spatial diffusion theory.

Perhaps one of the obstacles hindering, discouraging, and/or distracting researchers in this initiative is a lack of up-to-date urban hierarchy articulations; some that can be found are incomplete, being for only the highest tiers, whereas others are missing altogether (see [3]). Griffith and Li [5] were forced to sketch their own structures. Their US urban hierarchy is a revised version of one created by Yeates and Garner much earlier using 1970 data and released to the intellectual community around 1980, but never reworked thereafter until 2021. Dramatic metamorphic changes transpiring that could and should not go unheeded include the following: (1) Detroit’s precipitous plummeting from the top echelons; (2) noticeable descents by, for example, Baltimore, Pittsburgh, and San Francisco; and (3) a rapid ascent of cities like Atlanta, Dallas, and Phoenix. Meanwhile, in-progress rural-to-urban migration (re certainly throughout 2005-2024; [15]), resembling what took place in the US during the mid-1900s, spectacularly and continuously regulates and regenerates China’s modern-day urban hierarchy (e.g., [16]). Once more, extraordinary restructuring shifts in urban hierarchy ranks since 2000 (e.g., see [17]) that could and should not be disregarded include: (1) the northeastern regional cluster of cities (e.g., Dalian, Harbin, and Shenyang) suffering a coincidentally co-occurring downgrade from mid- to lower-tiers; (2) the unmistakable upward mobility of cities like Chengdu, Hangzhou, Nanjing, Shenzhen (especially), Wuhan, and Xi’an; and (3) the bewildering loss-of-prominence downward slump of, for example, Changchun, Dalian, Shijiazhuang (especially), and Wenzhou.

Griffith and Li [5] muster mandatory tabularized city data and apposite maps to configure just the top strata of the US and Chinese city systems, respectively, implanted in coterminous states and provinces, the geographic binning substructures for COVID-19 data collection; hence, their schema are biased urban hierarchy substitutes. Their objective was to bridge national trellises to a global city system edifice (e.g., [18,19,20]—but neither furnishes a world city network hierarchy diagram superior to the 2015 picture crafted by Taylor and Derudder) because the pandemic was worldwide, and these conduits were major gateways for the disease to gain entrance into the US and out of China. Next, they marshaled the two nationwide contagion and hierarchical spread instruments within the MESF paradigm in its space-time incarnation (i.e., MESTF). In other words, they constructed SWMs, generically denoted by **C**_H_, for the two hierarchies—where subscript H stipulates that matrix entries are direct hierarchical connections, adopting the matrix symbol **C**_s_ to renamed commonly unsubscripted SWM **C**, by obvious design, to denote contiguity connections—modified each in accordance with the MC formula [see expression (2)], and then extracted prominent eigenvectors affiliated with either positive or negative SA. Their findings, which demonstrate that incorporating hierarchical SA improves descriptions and forecasts, divulge the following (see Table 3): (1) although the commencement of a disease diffusion process means many areal units start by having a zero infection cases count (i.e., initially, the first disease appearance most likely is in a solitary location), SA rather than a more complicated zero-inflated model accounts for this irregularity; (2) contagion and hierarchical SA together account for a very sizeable percentage of geographic variability in the spread of COVID-19 across the coterminous US and mainland China; (3) a negative binomial may or may not be more appropriate than a Poisson probability model specification; and (4) one-day forward forecasts should be quite reliable (which may be sufficient for planning medical intercessions during the opening phases of a pandemic crisis).

In conclusion, the world is lacking justifiable constructed urban hierarchies, mostly because the few available ones are obsolete, in total or in part. Maintaining a portfolio of them for all countries—195 at last count—and the world is a utopian but formidable scholastic commitment, one rivaling the time and financial demands needed for disseminating and then sustaining computer software packages. After devising an inaugural urban hierarchy comes its debugging, and then its intermittent renovation, with these last two steps iteratively repeated every, say, five years or so, ad infinitum. A fundamental and worthwhile benefit of this perpetual endeavor is the explicit quantitative inclusion of hierarchical SA in geospatial data analyses, which should track a similar trajectory as that over the past half century for incorporating contiguity SA in linear regression models, a venture that ultimately switched them to spatial regression models. Such a new equation term enriches understanding, interpretations, and prediction capabilities, all indispensable to advancing the spatial sciences in general, and spatial epidemiology in particular.

## 5. Challenges

Given the prior discussion, sensibly couched in other literature sources, the two most obvious challenges put forth and advocated by this perspective are the following: (1) cataloging positive–negative SA mixtures for geographic distributions of procured ailment cases, particularly ones stemming from common environmental factors (i.e., human-environment interactions), and promoting customary insertion of these interminglings into spatial statistical techniques; and (2) catapulting geographic hierarchy SA into a mainstream integral facet status for routinely specifying spatial regression model and other descriptive geospatial equations. Both points allude to advancing a true or validity amelioration of spatial analysis procedures.

An additional present-day challenge is to go beyond conceding positive–negative SA mixtures, establishing cogent rationales for each of these two disease data components as a regular and systematic exercise. For example, the *Anopheles gambiae* complex of mosquitoes seeks habitats with the same attributes, ingraining positive SA, while simultaneously competing for territory, entrenching negative SA. In addition, human cancer cases may cluster in geographic space, projecting positive SA—possibly as a Schelling model type manifestation (e.g., households with similar lifestyles tend to congregate their residencies in relatively homogeneous domiciliary neighborhoods spatially differentiated amongst by family socio-economic/demographic attributes)—whereas screening people/animals with undiagnosed cases may have an awareness distance decay element inculcating negative SA (i.e., relatively high rate zones flanking mediated elective treatment induced relatively low rate zones).

A transparent technical ultimatum is omitted variable bias (i.e., the estimated coefficient of one or more covariates in a regression equation is biased—namely, the arithmetic mean of its sampling distribution statistics fails to equal its population parameter value—by exclusion of a relevant explanatory variable from its specification; see [21]), which arises in the positive–negative SA mixture context because an extant negative SA term is absence from an equation. Table 4 listings (i.e., comparative deviance statistics) uphold the previous declaration that positive SA alone is responsible for the biggest share of geographic variance inflation. Meanwhile, although discarding positive SA can inject acute, forsaking negative SA can introduce obfuscating omitted variable bias into parameter estimates calculated with a spatial regression technique (e.g., three of the six specimen intercept estimates fall outside of their corresponding 95% confidence interval; sequential confidence interval approximate z-scores are as follows: 0.4 (Poisson), 0.8, 3.0, 5.3, 0.0 (normal linear), −25.2)); standard errors are modestly prone to such bias, too.

This missing variables’ specification error scenario is especially relevant here because it underscores the inherent heterogeneity of geospatial disease data, with positive SA exacerbating it through variance inflating hot and cold spot manifestations [a la local indicators of spatial association (LISA; [22]), a popular spatial epidemiology tool], and negative SA moderating it by dispersing contrasts across a geographic landscape. Certainly, employing methods that take into account geospatial heterogeneity via accurate nonconstant regression mean responses (i.e., fitted/predicted values) should enhance future SA research in environmental health, which further emphasizes the importance of the omitted variables relationship with negative SA.

For historical reasons, epidemiology is the health sciences subfield attraction here. Another medical area whose SA attentiveness is on the rise is computerized visualization of the interior of a body for clinical analysis and therapeutic intervention as well as sequential 2-D cross-section scanning slices emulating a 3-D representation of the function of some organs or tissues. It is a context encumbered with SA that is once again inspiring some in the next generation of spatial analysts (e.g., [23]).

The innovative notion of urban hierarchy SA, the other exigent perspective issue explicated in this paper, builds upon a (near-)planar graph theory success story that now has earned a moderately protracted history of stability across individual implementations, primarily because of the pervasive convenient accessibility of advanced GISs and their user-friendly built-in spatial statistical tools. Today, long time horizons may require manual supervision of non-hierarchical SWM construction corrections; however, short time horizons (e.g., five- or ten-year periods) rarely do. The parallel for urban and other geographic hierarchies is that they often are in a stage of constant flux, and, accordingly, command adaptive learning modifications on an unending basis. Like their contiguity cousins, one pending task is designing a universally available executable automated gadget that converts input criteria metrics into output hierarchical SWMs, radically streamlining the tedious and time-consuming chore of supervising a manual composing of them.

## 6. Future Directions

By its very core subject, the preceding section touches upon certain imminent research directions. Intriguingly, several add-on problems immediately come to mind that can augment its implied agenda. Both derive from an obligation and ability to decipher SA, regardless of its ilk, in some meaningful way. On the one hand, exposing concealed SA, which is a systematic non-stochastic correlational and redundant information data trait, argues that omitted substantive attribute variables are compromising a descriptive geospatial equation as well as estimation of its parameters. A typical rection to this conundrum is for an analyst to search for these missing variates or their surrogates, a tactic encouraged decades ago vis à vis regression residual examination; another reaction is to create proxies (e.g., ESFs) to assuage any collateral damage (e.g., [24]), a maneuver incited in mixed models by inclusion of an artificial random effects term. Griffith and Li [5] elucidate this strategy, carefully scrutinizing relevant operationalized attribute variables and finding that the pairs of China and US SA components appear to be at least partial substitutes for per capita living space, a ratio of non-agricultural to agricultural population, and a male-female ratio, for China, and the retail income percentage, the influenza death rate, and the 85-year-old population percentage, for the US. Each of these potential equation-absent covariates has a genuine logical bond with the observed COVID-19 death counts, which these co-authors speculatively spell out.

**Figure 2:**
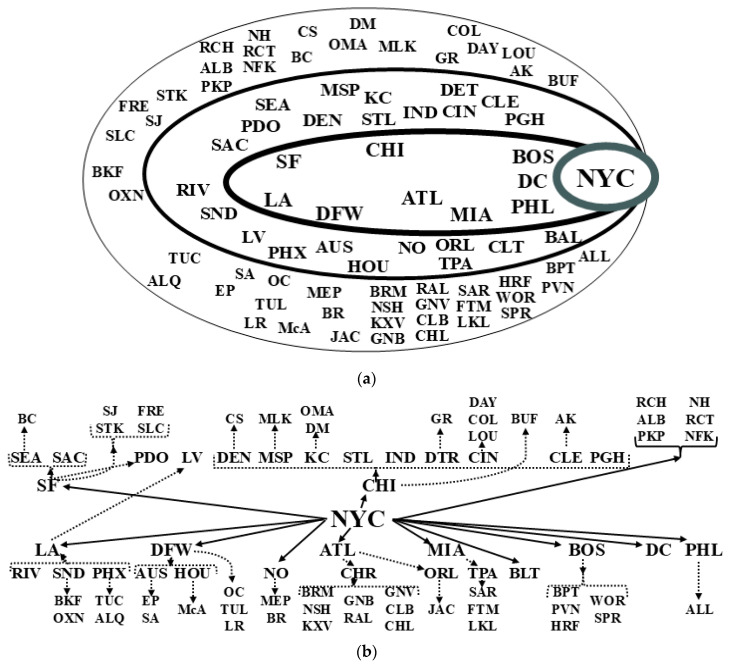
A redrafting of the latest US urban hierarchy rendition (after [3]), top 84 metropolitan areas (Akron—AK; Albany—ALB; Albuquerque—ALQ; Allentown—ALL; Atlanta- ATL; Austin—AUS; Bakersfield—BKF; Baltimore—BAL; Baton Rouge—BR; Birmingham—BRM; Boise City—BC; Boston—BOS; Bridgeport -BPT; Buffalo—BUF; Charleston, SC—CHL; Charlotte—CHR; Chicago—CHI; Cincinnati—CIN; Cleveland—CLE; Colorado Springs—CS; Columbia—CLB; Columbus—COL; Dallas-Ft. Worth—DFW; Dayton—DAY; Denver—DEN; Des Moines—DM; Detroit—DET; El Paso—EP; Fresno—FRE; Ft. Myers—FTM; Grand Rapids—GR; Greensboro—GNB; Greenville—GNV; Hartford—HRF; Houston—HOU; Indianapolis—IND; Jacksonville—JAC; Kansas City—KC; Knoxville—KXV; Lakeland—LKL; Las Vegas—LV; Little Rock—LR; Los Angeles—LA; Louisville—LOU; McAllen—McA; Memphis—MEP; Miami—MIA; Milwaukee—MLK; Minneapolis-St. Paul—MSP; Nashville—NSH; New Haven—NH; New Orleans—NO; New York—NYC; Norfolk—NFK; Oklahoma City—OC; Omaha—OMA; Orlando—ORL; Oxnard—OXN; Philadelphia—PHL; Phoenix—PHX; Pittsburgh—PGH; Portland, OR—PDO; Poughkeepsie—PKP; Providence—PVN; Raleigh—RAL; Richmond—RCH; Riverside—RIV; Rochester—RCT; Sacramento—SAC; Salt Lake City—SLC; San Antonio—SA; San Diego—SD; San Francisco—SF; San Jose—SJ; Sarasota—SAR; Seattle—SEA; Springfield—SPR; St. Louis—STL; Stockton—STK; Tampa—TPA; Tucson—TUC; Tulsa—TUL; Washington, DC—DC; Worcester—WOR.) in 2020. Top (**a**): the four nested tiers. Bottom (**b**): the system articulation.

On the other hand, analogous to the MESF-informed explanation of contiguity SA (i.e., global, regional, and local clusters of relatively similar (positive SA) or contrasting (negative SA) attribute values), an eloquent interpretation of hierarchical SA conceives its components as being tier-wide, moderate sized tier-spanning subsets, and shallow collections of (near-)abutting tiers. Figure 2 portrays the upper echelons of the US urban hierarchy formulated by Griffith [3], but in a different layout, and the infrastructure underlying the US states pyramid utilized in Griffith and Li [5]. Figure 2a is a drawing in keeping with the appealing format used by Taylor and Derudder, whereas Figure 2b adheres to the blueprint of flowcharts. Cooperatively, these two visuals coherently convey urban system structuration. Their key weakness is, in keeping with Yeates and Garner, that they incorporate only 84 of the 384 2020 US metropolitan statistical area cities. Their auxiliary frailty is that, like the presentational approach of Yeates and Garner, for economy of space and other parsimony considerations, they hypothetically collapse the fourth and fifth tiers into a single group, perchance obscuring a few hierarchical linkage details. Exploratory data analyses insinuate that this allegation is questionable; the set of 51 cities is consistent with a unified layer. An obvious future direction is the completion of a seminal total US urban system hierarchy, most likely with six levels, including two beyond Figure 2, each containing about 125 and 175 cities, respectively.

**Figure 3:**
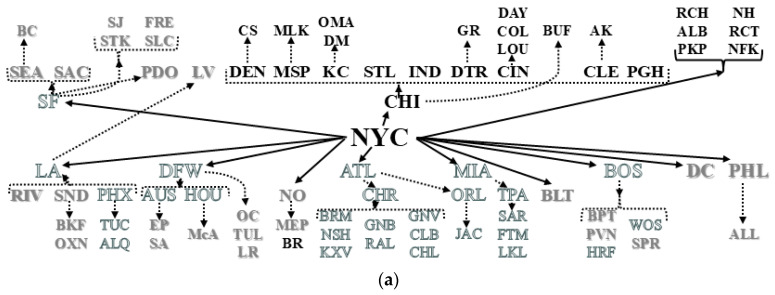

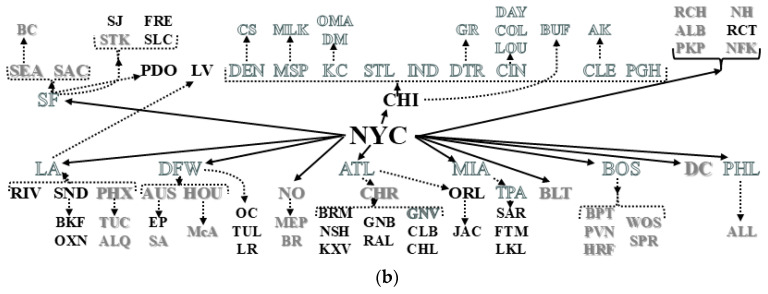
Eigenvectors of the extreme eigenvalues for the MESF-adjusted SWM **C**_H_; a tertile classification in which black, gray, and outlined white, respectively, denote relatively high, near-zero, and low values. Top (**a**): the maximum positive hierarchy SA eigenvector, **E**_1_, portrayal. Bottom (**b**): the maximum negative hierarchy SA eigenvector, **E**_n_, portrayal.

**Figure 4:**
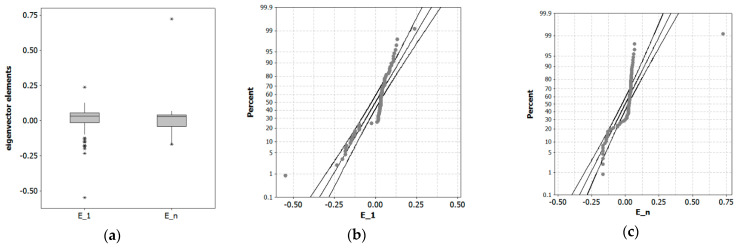
US urban hierarchy (n = 84) extreme eigenfunction eigenvectors. Left (**a**): eigenvector element boxplots (asterisk denotes outliers). Middle (**b**): normal quantile plot of **E**_1_ vector elements. Right (**c**): (**b**): normal quantile plot of **E**n vector elements.

Figure 3 shows two of 83 distinct eigenvector mappings onto the Figure 2b trellis (the 84th is a constant vector proportional to **1**, the intercept covariate in regression equations). Figure 3a embodies the maximum positive hierarchical SA achievable with the posited US urban system **C**_H_ SWM, whereas Figure 3b visualizes its opposite extreme, the maximum negative hierarchical SA. This former case yields MC = 1.06 and GR = 0.95 (i.e., a zero SA inference), whereas this latter case yields MC = −0.79 and GR = 3.41, two pairs of index values, the former conflicting and the latter disturbingly compatible (i.e., 3.41 is alarmingly greater than an anticipated value of about 2), hinting at the presence of muddled teledependency. Figure 4 spotlights eigenvector element anomalies that almost certainly contribute to this uncertainty of meaning. The MC values seem insightful; the GR values seem to beckon numerary perplexity, overstating the influences of the respective solo outliers. Even discounting a solitary egregious data corruptor per vector, each frequency distribution still is ill-behaved, lacking a preferred symmetry and, better yet, bell-shaped profile. The MC is somewhat insensitive to these distortions from such a commonly agreed upon paragon of data analytics. The GR is not because it accents both numerical aberrations (e.g., the isolated eccentricities Figure 4a–c reveal) and excessive numbers of structural neighbors (e.g., because it sits at the top of the US urban hierarchy, NYC connects to all n−1 other places in its national city network by default, assigning it 83 neighbors in a system with an arithmetic average of 4.64 such ties per metropolitan area).

Ushered by the MESF tradition of mapping contiguity SA eigenvectors to help decode the information they impart, Figure 3 conjures several noteworthy annotations. First, the positive hierarchical SA elements differentiate different branches or tiers of an urban hierarchy: similar vector element values cluster in concentrated hierarchical limbs or across hierarchical strata. Figure 3a chiefly discloses the Chicago conurbation, while discriminating between the upper and lower strata for the rest of the US. In contrast, Figure 3b unveils a more fragmented treillage, by both strata and limbs, an anticipated outcome when the aim is to maximize contrasts. The needed future direction here is a more in-depth and comprehensive examination of the entire modified matrix **C**_H_ eigenfunction spectrum.

## 7. Remarks, Conclusions, and Implications

This scholarly writing presents my commentary, remarks made in more of an editorial fashion (i.e., persuasive verbal reasoning seeking to encourage a geospatial audience to consider adopting the posited controversial perspective and then act by favorably amending their attitude to heed its two advisements), about two specific emerging trends and issues in geospatial environmental health that I have and continue to champion, namely finally recognizing and exploiting positive–negative SA mixtures in geo-tagged disease data, and efficacy of the urban hierarchy in understanding and modeling, especially for forecasting purposes, disease proliferation. Its focus is on certain recent developments, trends, and debates revolving around this topic. Unlike most perspective pieces in which new findings fail to appear, it pensively resembles original research articles only in that it reports a few (e.g., Table 2 and Table 4, and Figure 3 and Figure 4) of this type of fresh results. In doing so, it synthesizes resolutely chosen existing research and offers novel personal interpretations and convictions, hopefully provoking innovative discussion and action, highlighting literature gaps that should then become more conspicuous, hence suggesting future directions for study. Moreover, the viewpoints expressed in this essay are ones about which I remain adamantly passionate, whether or not they are themes fervently shared by other spatial scientists. My evidence-based expert and informed opinion views derive from intuition, theoretical and substantive knowledge, technical concept formation experiences within the academy, years of disciplinary application-oriented practice, and emergent literature themes such as machine learning utilization benefits—a promising avenue for SA research involving environment health—for spatial epidemiology (e.g., [25]). Promulgating these outlooks compels me to cite multiple self-authored publications already broadcasting my perspectives and their supporting arguments, although the percentage (i.e., 29.2%) of such references for this paper is not excessive (see [26]), a bibliographic assemblage diversified by its interlacing with some of the latest editions in its relevant literature. Obviously, another specialist may well compose such a perspective treatise about either different environmental health subjects, or the same two troubling matters but with a different emphasis. Normative acuities and consensuses seldom converge instantaneously within a large body of thinkers, a lesson learned from, for example, the acclaimed Delphi method.

The two central issues this paper discusses—negative and hierarchical SA—encompass great promise for impacting real-world spatial statistical applications like disease mapping and environmental health policy. For example, correcting omitted variable bias ensures that regression coefficient estimates are unbiased, statistically consistent, and more reliable, rendering better predicted values, which translate into better disease risk and forecasting maps. The same can be said about hierarchical SA, as the Griffith and Li [5] COVID-19 diffusion paper illustrates. Table 2 is a deafening declaration of this latter assertion.

**Figure 5:**
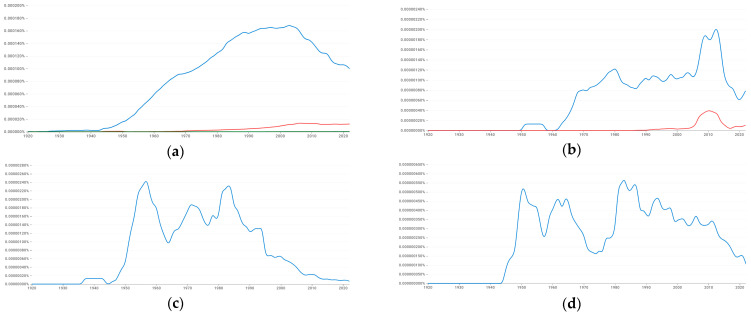
Time series usage of the terms autocorrelation (blue), “spatial autocorrelation” (red), and “negative spatial autocorrelation” (green). Source: a Google online search engine that charts the yearly count frequency of these alphanumeric strings found in printed sources published between 1500 and 2022 in Google’s English, Chinese, French, and German text corpora. Top left (**a**): English. Top right (**b**): Chinese. Bottom left (**c**): French. Bottom right (**d**): German.

**Figure 6:**
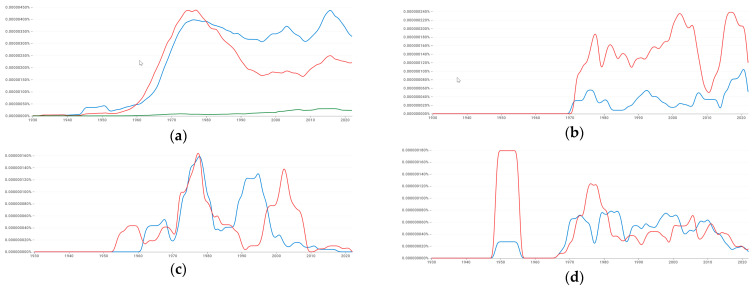
Time series usage of the terms “urban hierarchy” (blue), “central place theory” (red), and “city hierarchy” (green). Source: a Google online search engine that charts the yearly count frequency of these alphanumeric strings found in printed sources published between 1500 and 2022 in Google’s English, Chinese, French, and German text corpora. Top left (**a**): English. Top right (**b**): Chinese. Bottom left (**c**): French. Bottom right (**d**): German.

Meanwhile, prefacing a summary of conjectures and refutations tendered in this paper is the fact that, for millennia, science was oblivious to the actual autocorrelation phenomenon itself, which has characterized the Earth for billions of years, let alone SA or negative SA. In a great vault forward, Laplace became tacitly aware of autocorrelation in a time series context during the 1800s (with regard to his inspections of barometric pressure systematically moving across Europe). But this word did not first enter the scientific nomenclature until roughly 1920 (Figure 5), with its analysis not becoming a fashionable pursuit until around 1970 when Box and Jenkins popularized it. Meanwhile, SA as a jargonistic phrase had its debut decades later, prior but closer to 1970, whereas negative SA, besides mentioning a checkerboard for pedagogic intentions, premiered around 1976 (Figure 5a). In the meantime, unlike the lexicon of autocorrelation terminology, explicit urban hierarchy ideas date back to the near-dawn of colonialism (i.e., succeeding the Phoenicians cultivating settlements along the Mediterranean Sea coastline), an era when a plethora of autonomous city-states gave way to a spatial organization necessitated by the managerial encumbrances of post-conquest. The Roman Empire, Chinese Qin Dynasty, Mayan kingdom, and Indus Valley civilization coupled with its ensuing Maurya and Gupta Empires all basically concocted a strain of urban hierarchy that persists to this day. However, implanting it into modern data analytic work actually did not occur until approximately 1930 (Figure 6), pragmatically coinciding with the discovery of central place theory separately by Christaller and Lösch (Figure 6a–d attest to a close covariation over time between these two similar but distinct constructs), a conceptualization that propelled this idea to the forefront of geographical analysis thought. This perspective critique includes outlined plans enabling judicious usage of these two instruments to better serve humanity henceforth.

This paper also demarcates advisable groundbreaking research prospects. Going beyond Cliff and Ord’s crucial contributions establishing SA as a critical concept in spatial statistics, GISs, spatial analysis, and the geospatial information sciences, cutting edge enquiries need to probe negative SA in general, and positive–negative SA mixtures in particular. One aspiration of these endeavors must be credible explanations of why negative SA should be present; keeping in mind that spatial competition is one of its prime sources should prove helpful. Furthermore, Batty, arguably the Father of city science, devotes an entire chapter (i.e., 5: Hierarchies and networks) of his *The New Science of Cities* book to urban hierarchies, underlining the following important properties: time-invariant city-size orderliness (e.g., the rank-size rule); vertical distributional regularities (e.g., a geometric progression relating counts of cities lodging in each of a sequence of specified levels); urban hierarchical volatility (e.g., cities reshuffle their relative ordering positions with the passing of time); spatial competition and geographic flows, inputs into urban hierarchy formation; teledependency patterned spatial diffusion outputs from urban hierarchy channelings; and, lateral and adjoining tier interlockings of cities. All of these research frontiers interleave with implications already expounded upon expressly in the preceding section. The fundamental takeaway from this paper is a prodigious paradigm shift, a marked change in the collective mindsets and applications of spatial epidemiologists, for example, when specifying spatial regression equations to describe either environmental health data, or a publicly transparent geographic diffusion of diseases. Table 1 and Table 4 combined with Griffith and Li [5] furnish testimony for this belief.

## Figures and Tables

**Table 1:** Spatial regression summary statistics for SA mixture components.

Disease	Auto-Normal SARMA			GLM MESF			
	${\hat{\rho }}_{SAR}$	r	$-{\hat{\rho }}_{MA}$	ESF_p_	Pseudo-R^2^	ESF_n_	Pseudo-R^2^
cholera bacteria	0.904	0.83	−0.317	12 of 29	0.841	2 of 29	0.029
West Nile virus	0.972	0.92	−0.839	11 of 65	0.204	3 of 89	0.049
tuberculosis bacteria	−0.365	0.84	0.878	5 of 18	0.247	5 of 25	0.056
chikungunya virus	0.966	0.92	−0.869	3 of 13	0.548	3 of 18	0.009
Malaria parasite	0.729	0.97	−0.833	6 of 29	0.182	10 of 42	0.116
COVID-19 virus	0.475	0.92	0.308	5 of 8	0.895	3 of 12	0.095

Note: GLM and SAR, respectively, denote generalized linear model and simultaneous autoregressive; a moving average (MA) parameter sign is the opposite of its true SA nature; and, none of the six ${\hat{\rho }}_{SAR}$-${\hat{\rho }}_{MA}$ correlations are problematic (even r = 0.97).

**Table 2:** Google Scholar search results (29 November 2024).

Year	2020	2021	2022	2023	2024
COVID-19 spread	9570	15,600	13,700	9930	4640
COVID-19 spread + urban hierarchy	3	7	6	2	1

**Table 3:** Joint contagion-hierarchical SA summary results from Poisson regression.

	Fitting		Extrapolataing		
Statistic	China	US	Forecasting Day	China R^2^	US R^2^
deviance statistic (1 is desired)	1.25	8.58	+1	0.992	0.576
pseudo-R^2^	0.998	0.896	+4	0.002	0.510
${\hat{\pi }}_{\mathrm{zero}\mathrm{inflation}}$ probability	0.008	0.022	+8	0.005	0.227

**Table 4:** Omitted variable investigations via the pure SA descriptor intercept term, Table 1 data.

Disease	Constant Only			Constant + ESF_p_			Constant + ESF_p_ + ESF_n_		
	Intercept	se	dev	Intercept	se	dev	Intercept	se	dev
cholera bacteria ^†^	1.795	0.042	11	0.874	0.083	2	0.844	0.084	2
West Nile virus *	−4.181	0.016	12	−4.290	0.018	5	−4.306	0.019	4
tuberculosis bacteria *	−3.920	0.017	17	−4.163	0.023	10	−4.236	0.024	6
chikungunya virus *	−1.951	0.017	74	−2.175	0.020	23	−2.292	0.022	16
malaria parasite ^‡^	0.700	0.071	<1	0.700	0.064	<1	0.700	0.065	<1
COVID-19 virus *	−4.165	0.007	2183	−5.624	0.015	336	−5.297	0.013	118

Note: se and dev, respectively, denote standard error and deviance statistic (ideal value is 1). ^†^ Poisson regression; ^‡^ normal linear regression; * binomial regression.
